# Fast MRI Reconstruction Using Deep Learning-based Compressed Sensing: A Systematic Review

**Published:** 2024-04-30

**Authors:** Mojtaba Safari, Zach Eidex, Chih-Wei Chang, Richard L.J. Qiu, Xiaofeng Yang

**Affiliations:** 1 Department of Radiation Oncology and Winship Cancer Institute, Emory University, Atlanta, GA 30322, United States of America,

**Keywords:** Compressed sensing (CS), magnetic resonance imaging, MRI reconstruction, fast MRI, MRI acceleration

## Abstract

Magnetic resonance imaging (MRI) has revolutionized medical imaging, providing a non-invasive and highly detailed look into the human body. However, the long acquisition times of MRI present challenges, causing patient discomfort, motion artifacts, and limiting real-time applications. To address these challenges, researchers are exploring various techniques to reduce acquisition time and improve the overall efficiency of MRI. One such technique is compressed sensing (CS), which reduces data acquisition by leveraging image sparsity in transformed spaces. In recent years, deep learning (DL) has been integrated with CS-MRI, leading to a new framework that has seen remarkable growth. DL-based CS-MRI approaches are proving to be highly effective in accelerating MR imaging without compromising image quality. This review comprehensively examines DL-based CS-MRI techniques, focusing on their role in increasing MR imaging speed. We provide a detailed analysis of each category of DL-based CS-MRI including end-to-end, unroll optimization, self-supervised, and federated learning. Our systematic review highlights significant contributions and underscores the exciting potential of DL in CS-MRI. Additionally, our systematic review efficiently summarizes key results and trends in DL-based CS-MRI including quantitative metrics, the dataset used, acceleration factors, and the progress of and research interest in DL techniques over time. Finally, we discuss potential future directions and the importance of DL-based CS-MRI in the advancement of medical imaging. To facilitate further research in this area, we provide a GitHub repository that includes up-to-date DL-based CS-MRI publications and publicly available datasets - https://github.com/mosaf/Awesome-DL-based-CS-MRI.

## 1 Introduction

Magnetic Resonance Imaging (MRI) is a highly effective medical tool that produces high-quality images of soft tissues in the body. It is widely used in the lesion prognosis and diagnosis, radiation treatment planning, and follow-up examination. However, the Lancet Oncology Commission recently highlighted a severe shortage of MRI and other medical imaging technologies in low-income and middle-income countries (Hricak et al., 2021). This shortage has resulted in 2.5 million deaths worldwide. The installation of MRI scanners remains low globally, with only 7 MRI scanners per million people installed as of 2020 (Y. Liu et al., 2021). This is primarily due to the high cost of installation, operation, and maintenance. Besides, the daily throughput is limited by the long acquisition time required for each MRI scan. The long wait time reduces the number of patients that can be seen in a given day (Murali et al., 2023). Additionally, it increases the likelihood of voluntary and involuntary patient movements (Safari et al., 2023b), causing motion artifacts that affect the accuracy of the images produced. The estimated cost of motion artifacts induced by patient movements is around $364,000 per scanner annually (Slipsager et al., 2020).

**Figure 1:**
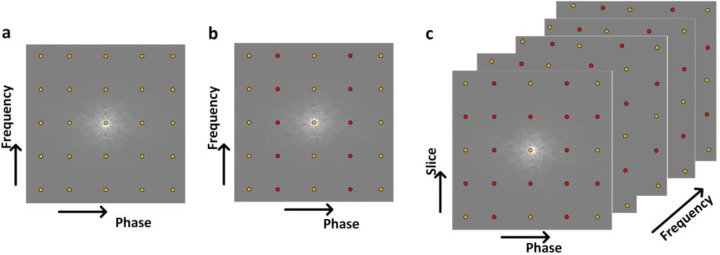
The schematic diagrams of the k-space sampling pattern with a Cartesian trajectory are illustrated for (a) fully sampled data and (b) undersampled 2D MRI data in phase encoding direction with acceleration rate (R) 2 as well as (c) undersampled 3D MRI in both slice and phase encoding direction with *R* = 2. The yellow and red circles indicate sampled and skipped data during the data acquisition.

MRI requires densely sampled k-space to avoid violating the Nyquist criteria, which results in longer acquisition times for high-resolution images. To reduce the imaging time, *k*-space can be undersampled in the phase encoding direction by increasing the spacing between *k*-space lines and, therefore, covering the field of view in a shorter amount of time, as illustrated in Figure 1 for a Cartesian trajectory. Compressed sensing (CS), also known as compressive sensing or compressive sampling, is a method that aims to reconstruct fully-sampled k-space from undersampled k-space by exploiting the images’ sparse representation in a transform domain such as Cosine and Wavelet (Haldar et al., 2010). The CS algorithms optimize a cost function (1) to iteratively reconstruct the image. However, CS algorithms are unable to completely reconstruct the high-frequency texture content of images (Ravishankar and Bresler, 2010), limiting them to acceleration factors between 2.5 and 3 (Guo et al., 2021a). In addition, these iterative techniques inevitably increase reconstruction time.

(1) $$\underset{x}{\text{min}}=\|\Psi \text{x}{\|}_{2}s.t.{\left\|{\text{y}}_{\Omega }-{\text{E}}_{\Omega }\text{x}\right\|}_{2}^{2}<\epsilon ,$$

where ${\text{E}}_{\Omega }\in {\mathbb{C}}^{M\times N}\to {\mathbb{C}}^{M}$ given *N* > *M* is the encoding operator. It is composed of a coil sensitivity map, a Fourier transform, and a sampling map with the specified pattern $\Omega \cdot {\text{y}}_{\Omega }\in {\mathbb{C}}^{M}$, $\text{x}\in {\mathbb{C}}^{N}$ and *ϵ* are the undersampled *k*-space measurement, fully sampled image, and the threshold controlling the reconstruction fidelity, which is roughly equal to the expected noise level, respectively.

**Figure 2:**
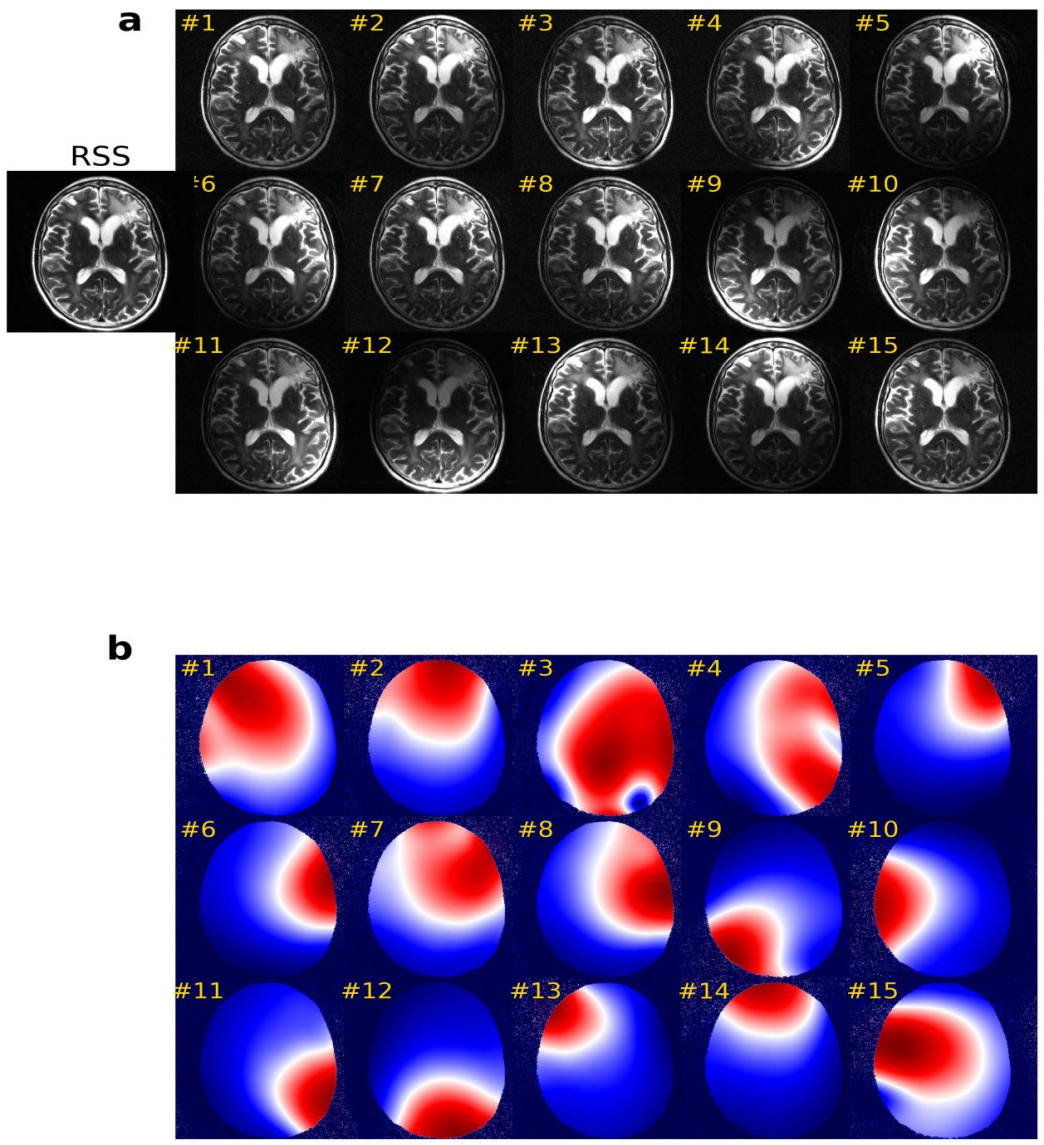
(a) Images and (b) sensitivity maps, estimated by the ESPIRiT approach (Uecker et al., 2014), are illustrated for the first 15 receiver coils. The root sum of squares (RSS) indicates combined weighted receiver coil images by the corresponding sensitivity maps.

Parallel imaging is another approach to reduce image acquisition time. Multiple receiver coils are placed in different positions within the scanner to independently collect a portion of the *k*-space data(Deshmane et al., 2012; Larkman and Nunes, 2007). Each coil is most sensitive to the area closest to it (shown in Figure 2a) with the sensitivity relationship encoded in the form of sensitivity maps (shown in Figure 2b). The final image is generated by combining the individual images obtained from different coils by taking their root sum of squares (RSS) and weighting them by the corresponding sensitivity maps.

Deep learning (DL) algorithms have garnered significant interest in CS-MRI applications. These DL models have demonstrated superior reconstruction performance at a higher acceleration rate compared to traditional non-DL-based CS models (Mardani et al., 2018; Qin et al., 2018). In light of the rapid advancements in this field, we conduct a systematic review, encapsulating the latest developments in state-of-the-art DL-based CS-MRI models. Table 1 provides a concise summary of the most relevant survey articles, revealing technical differences compared to our review. We also note that significant progress has been made in DL-based CS-MRI over the last 2 years, especially with growing interest in diffusion models and the DC layer, so our review better captures current research directions.

**Figure 3:**
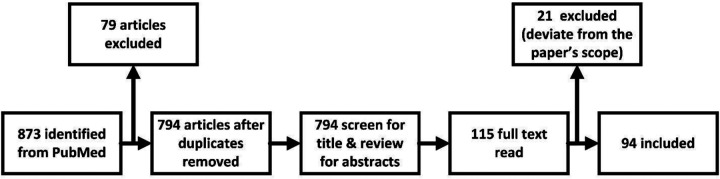
Flowchart of our study selection process.

The PubMed database was meticulously searched on February 1st, 2024, using the terms “deep learning reconstruction”, “fastMRI”, “unrolled optimization”, “MRI reconstruction”, and “MRI acceleration” for articles published from February 2024 to January 2016. Relevant studies were carefully screened by title and abstract content. Of the 873 publications identified by PubMed, 94 articles were included. Figure 3 illustrates the entire literature screening and selection process.

### 1.1 k-space trajectories

**Figure 4:**
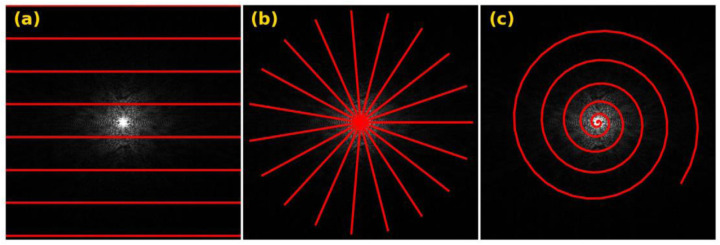
An exemplary k-space with overlaid (a) Cartesian, (b) radial, and (c) spiral trajectories illustrated.

Various trajectories have been developed in MRI for traversing k-space, including Cartesian, spiral, radial, and random trajectories. The Cartesian trajectory, as depicted in Figure 4a, consists of parallel lines equidistant from each other, with each line representing a frequency-encoding readout. The image can be reconstructed using a fast Fourier transform, but each line requires a separate RF pulse, prolonging imaging time.

The radial trajectory, first used by (Lauterbur, 1973) and shown in Figure 4b, consists of spokes radiating from the center, with an oversampling center in k-space that makes it robust to motion artifacts (Maclaren et al., 2013). However, undersampling in the azimuthal direction increases streak artifacts (Xue et al., 2012).

The spiral trajectory shown in Figure 4c was introduced to decrease the MRI acquisition time. It starts at the center of the k-space and spirals outward, similar to radial sampling, and is robust to motion artifacts. However, hardware limitations restrict imaging efficiency and increase image blurring.

### 1.1 Sampling patterns

**Figure 5:**
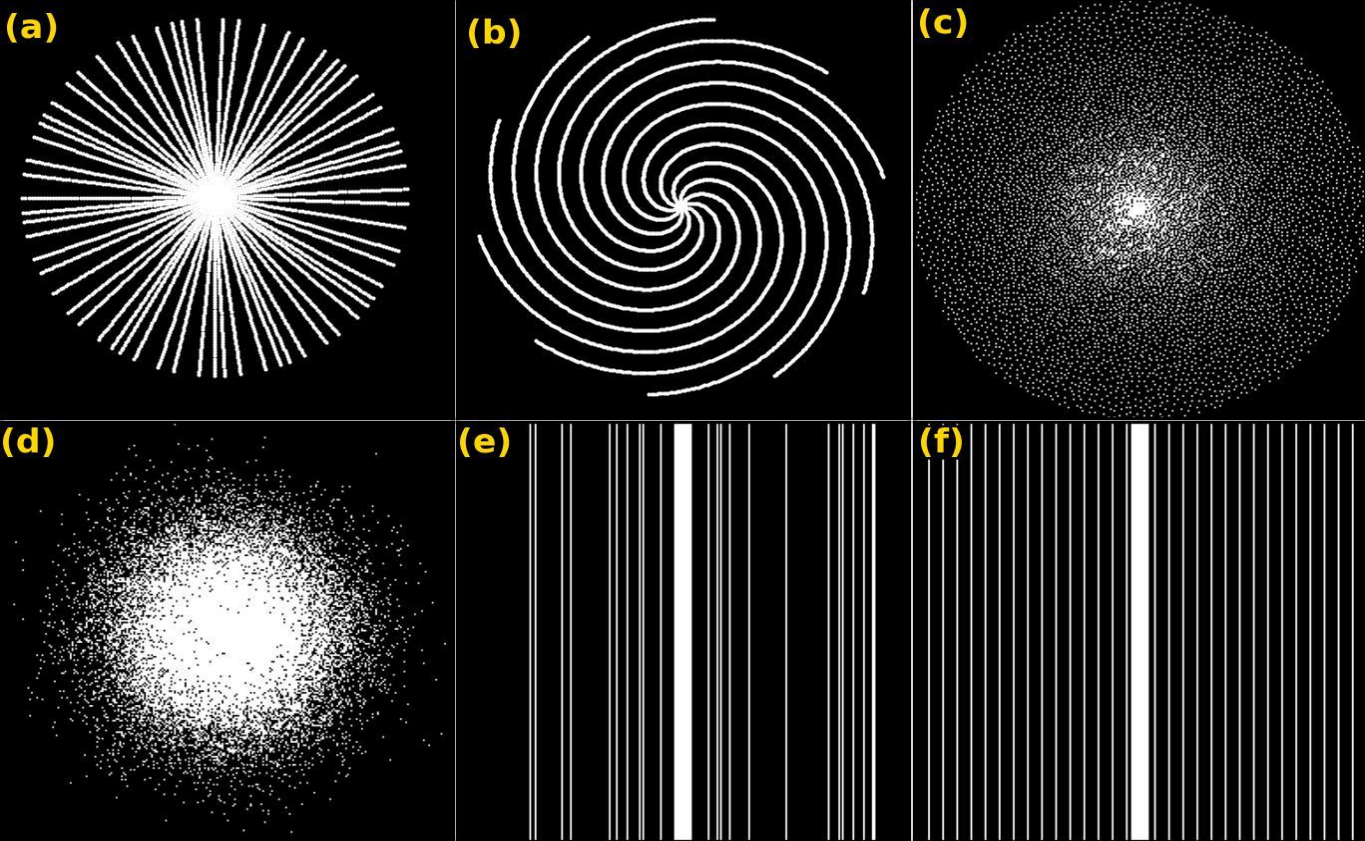
Widely used sampling pattern for k-space undersampling - (a) radial, (b) spiral, (c) Poisson disk, (d) Gaussian, (e) random Cartesian, and (f) equidistant Cartesian.

To speed up the process of capturing images, a pattern or mask, denoted as **Ω** in (1), is utilized to sample k-space. Numerous sampling patterns have been introduced for data acquisition techniques including Cartesian (Safari et al., 2024), Poisson (Slavkova et al., 2023), Gaussian (C. Hu et al., 2021), radial (Terpstra et al., 2023), and spiral patterns. Figure 5 illustrates examples of the different sampling patterns. The Cartesian pattern is typically used for brain and knee data with a Cartesian k-space trajectory while we found the Poisson and Gaussian patterns to be primarily used to train self-supervised models as described in Section 3.4. On the other hand, radial and spiral patterns are mainly used for capturing myocardial and dynamic images that are more likely to have motion artifacts.

## 2 Deep learning

### 2.1 Convolutional neural network

Convolutional neural networks, also known as ConvNets, are a type of deep neural networks that are designed to analyze grid-like data such as images and speech (LeCun et al., 1995). They have gained widespread recognition after the success of AlexNet (Krizhevsky et al., 2012) and have since been used to achieve state-of-the-art performance in various medical image processing and analysis tasks. ConvNets typically consist of multiple layers, with each layer including a convolutional operator, batch normalization layers, nonlinear activation functions, and dropout layers. Nonlinear activation functions are used to facilitate the learning of complex functions. Finally, weight regularization and dropout layers are employed to mitigate overfitting. During convolution, trainable convolution kernels slide over the images to extract multiple feature maps called channels.

The network’s optimum parameters are computed using a backpropagation algorithm that calculates the gradient of the cost function with respect to the parameters in each layer. Batch normalization layers are crucial in training deep ConvNets to prevent vanishing and exploding gradients. In addition, residual blocks (He et al., 2016) are a popular choice for building advanced ConvNets due to their ability to prevent gradient vanishing and facilitate smoother error surfaces (Li et al., 2018). By incorporating skip-layer connections between input and output, residual blocks can help reduce the risk of local minima. Furthermore, when combined with (batch) normalization layers, residual blocks can effectively address the problems of vanishing and exploding gradients.

### 2.2 Network architectures

#### 2.2.1 U-Net

Several deep learning models with different architectures have been proposed to enhance the performance and generalization of ConvNets. Among them, U-Net, with its elegant design that utilizes skip connections between the encoder and decoder, is the best known architecture in computer vision (Ronneberger et al., 2015). It has been extensively exploited in different medical applications such as image synthesis (Han, 2017), segmentation (Dong et al., 2019), and registration (Balakrishnan et al., 2019). In recent years, U-net architectures have incorporated residual and attention layers as a backbone to increase the network’s depth and improve performance.

#### 2.2.2 Transformer

While ConvNets have been impressive in their results, they are limited by the local context of convolutional operations. To address this challenge, Transformers have emerged as a solution to capture global context (Dosovitskiy et al., 2020), often outperforming ConvNets. However, transformer models are fundamentally very complex and require many trainable parameters and large databases for training which can be a challenge in medical imaging. To mitigate these issue, various variations have been proposed, such as Swin Transformers (Z. Liu et al., 2021), Vision CNN-Transformer (Fang et al., 2022), and ReconFormer (P. Guo et al., 2023), which aim to reduce model size while improving or maintaining performance.

#### 2.2.3 Generative adversarial network

Generative adversarial networks (GANs) are implicit methods. Thus, they do implicitly attempt to minimize likelihood function nor attempt to learn latent representation. The GAN, initially introduced in 2014, consists of two networks, generative and discriminator (Goodfellow et al., 2020). The former is trained to generate artificial data samples to approximate the target data distribution, and the latter is simultaneously trained to distinguish the artificial data from real ground truth data. Thus, the discriminators encourage the generator to generate data samples with a distribution similar to the target distribution. Variations of GANs have been developed to perform tasks including image-to-image translation, such as conditional GAN (Mirza and Osindero, 2014), StyleGAN (Karras et al., 2019), CycleGAN (Zhu et al., 2017), and Pix2Pix (Isola et al., 2017). GANs are widely used in medical imaging for tasks such as image registration, image synthesis, and MRI image reconstruction (Quan et al., 2018; Shaul et al., 2020; Yang et al., 2017).

#### 2.2.4 Diffusion model

**Figure 6:**
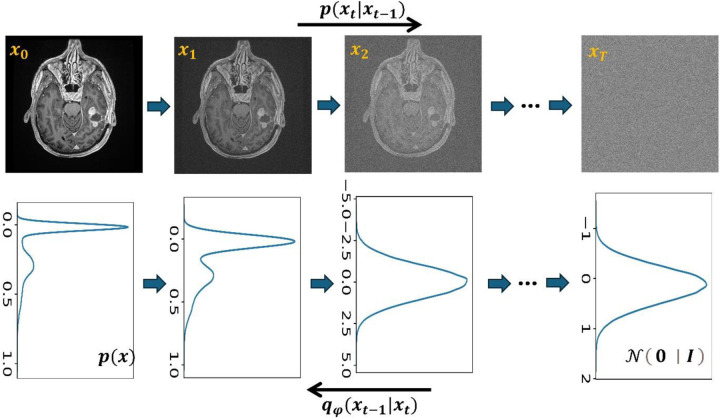
Forward and reverse diffusion processes. The first row indicates data in image space, and the second row indicates the corresponding data distribution. The forward diffusion process adds Gaussian noise in *T* steps in a controlled way to produce normal Gaussian noise in step *T*. The reverse diffusion process requires a model parametrized by *φ* to learn input *x*_0_ from noise-corrupted image *x*_*t*_ in a given step *t*.

The stable diffusion model, inspired by nonequilibrium thermodynamics, aims to simplify complex and difficult-to-calculate distributions using tractable ones like normal Gaussian distributions (Sohl-Dickstein et al., 2015). This model is comprised of two steps - the forward and reverse processes (Figure 6). During the forward process, Gaussian noise is added to the initial image *x*_0_ over *T* steps until the data at step *T* becomes normal Gaussian noise *x*_*T*_ = 𝒩(**0**, **I**). In the reverse process, the model learns to recover the original image *x*_0_ from its noisy version given at a step *t* ∈ (0, *T*] (Chan, 2024).

Stable diffusion models have been employed for medical imaging tasks such as denoising (Pan et al., 2023b), synthesis (Pan et al., 2023a), MRI distortion reduction (Safari et al., 2023b), and MRI image reconstruction (Chung and Ye, 2022; Güngör et al., 2023).

## 3 Deep learning for MRI reconstruction

**Figure 7:**
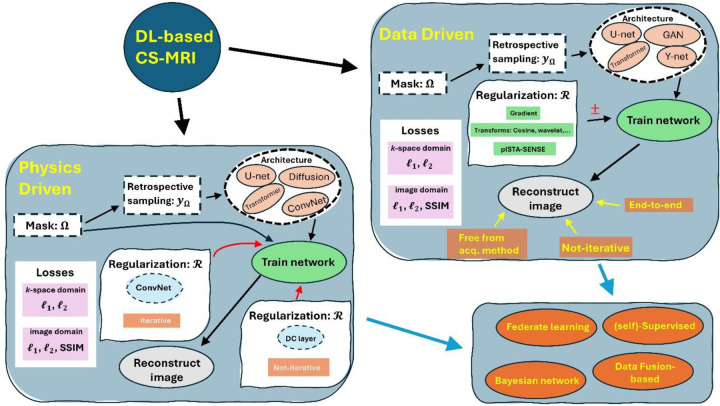
An overview of seven categories of DL-based CS-MRI methods.

The framework for DL-based CS-MRI can be divided into two main categories: data-driven and physics-driven models. Within these categories, there are two types of models: end-to-end and unroll. End-to-end models take in zero-filled k-space and output fully-sampled k-space. They typically utilize a regularization term listed in Table 2 to enforce the uniqueness of the reconstructed images. On the other hand, unroll models are more complex and further classified into two types: unroll optimization and closed-form models known for the “data consistency (DC) layer.” Unroll optimization models iteratively optimize the reconstruction process, while DC layer models use a closed-form equation to ensure data consistency. These models are utilized in various training scenarios, including federated learning and self-supervised training. For a comprehensive understanding of DL-based CS-MRI methods and their corresponding components and features, please refer to Figure 7.

**Figure 8:**
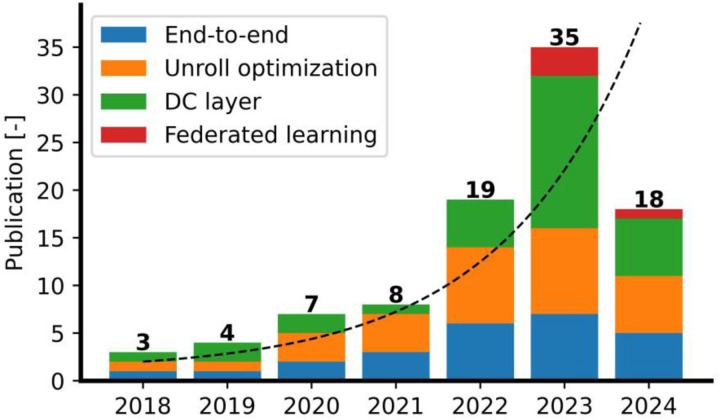
Overview of publications in DL-based CS-MRI over time. The dashed line indicates the general trend plotted using 1 + exp(0.62 × *t*) where *t* is defined in years.

Figure 8 shows a stacked chart of the number of publications since 2018 by category. The total number of publications has grown exponentially in recent years and interest in the DC layer method continues to increase while interest in unrolling optimization and end-to-end approaches remains consistent over time.

### 3.1 End-to-end models

DL end-to-end models are specifically designed to tackle the CS-MRI problem without enforcing any data acquisition model. To achieve this, these models rely on a neural network to accurately predict fully sampled data from undersampled data (B. Levac et al., 2023; Mardani et al., 2018; Yang et al., 2016). Additionally, these models are trained using various regularization techniques that help address the ill-posed inverse problem. Table 2 lists common regularization techniques utilized in these models.

The end-to-end approach employs the same baseline models that are used for image-to-image translation, such as the U-nets (Hyun et al., 2018; Xiang et al., 2018), Swin transformers (Huang et al., 2022), and GANs (Shitrit and Riklin Raviv, 2017; Zhao et al., 2023). However, they require a larger sample dataset than unrolling CS-MRI models and tend to predict images with synthetic data.. Table 3 provides a list of selected references that used end-to-end DL models to solve DL-based CS-MRI algorithms.

### 3.2 Unroll model

#### 3.2.1 Unroll optimization

Unroll CS-MRI models combine DL with a data acquisition model to solve an optimization problem iteratively. This optimization is given by Equation 2:

(2) $$\hat{x}=\text{argmin}\underset{\text{Data}\;\text{consistency}}{\underset{︸}{{\left\|y_{\Omega }-E_{\Omega }x\right\|}_{2}^{2}}}+\lambda \underset{\text{Regularization}}{\underset{︸}{ℛ(x)}}$$

In this equation, λ > 0 is a scalar regularization weight that balances between the data consistency and regularization terms. The data consistency term ensures that the reconstructed images $\hat{\text{x}}$ are similar to the given undersampled y_Ω_, thus enforcing data fidelity. The regularization term helps solve the ill-posed CS-MRI problem by imposing sparsity on the solution to guarantee the uniqueness of the reconstructed images, $\hat{\text{x}}$ (Donoho, 2006).

DL models, particularly ConvNets, are used heavily to learn the regularization term through an unroll training scheme. Similarly, the prior DL-based regularizes encode prior knowledge about the reconstructed images, such as sparsity. The unroll CS-MRI DL models have shown to outperform end-to-end methods using a network with a smaller number of trainable parameters (Aggarwal et al., 2018; Geng et al., 2023; Liu et al., 2022; Qiao et al., 2023; Qu et al., 2024).

However, the iterative nature of the unroll optimization method may increase the computation time during both training and inference steps as both the network’s weights and data consistency terms are simultaneously updated. Table 4 provides a list of references that used unroll optimization models to solve DL-based CS-MRI algorithms.

#### 3.2.2 Data consistency layer

The more popular approach is to train the unrolled models similarly to the end-to-end models as follows:

(3) $${\hat{x}}_{f_{\psi }}=\text{argmin}{\left\|x-f_{\psi }\left(x_{\Omega }\right)\right\|}_{1}+\lambda \underset{\text{Dota}\;\text{consistency}}{\underset{︸}{{\left\|y_{\Omega }-E_{\Omega }x\right\|}_{2}^{2}}},$$

where *f*_*Ψ*_ is a DL model that maps the undersampled input images *x*_*Ω*_ to reconstruct fully sample images *x*. The DL reconstruction and data consistency operate on the image domain and *k*-space domain, respectively. Although the DL part is trained without incorporating a priori information, the second term discourages the DL first part from updating the *k*-space parts that were not sampled (Schlemper et al., 2017). The closed form for (3) is as follows (Qin et al., 2018):

(4) $$\hat{x}=\{\begin{array}{cc} {\hat{x}}_{f_{\psi }}(k) & \text{if}\;k\notin \Omega \\ \frac{{\hat{x}}_{f_{\psi }}(k)+\lambda_{0}x_{\Omega }(k)}{\lambda_{0}+1} & \text{Otherwise} \end{array}$$

This closed form is a computational layer called the DC layer at the end of DL models. The DC layer is a crucial part of the CS-MRI DL model, playing an important role in reconstructing images (Cheng et al., 2021; Korkmaz et al., 2023). The DC layer allows for a flexible design of the DL model when it is added to U-net (Murugesan et al., 2021), transformers (Wu et al., 2023), stable diffusion model (Cao et al., 2024), and so on. Table 5 summarizes the DL-based CS-MRI trained under the DC layer framework.

### 3.3 Federated learning

Federated learning (FL) is a promising framework that enables the collaborative training of learning-based models across multiple institutions without the need for sharing local private data (Yang et al., 2019). The objective of FL models is to learn a global model by taking the average of local models (McMahan et al., 2017) or by ensuring the proximity of local models to the global model (Li et al., 2020). When applied to MRI image reconstruction, the FL offers unique advantages tailored to the specific challenges and requirements as follows:

- MR images often contain sensitive patient information that needs to be protected. FL enables MRI models to be trained directly on the devices where the images are acquired, without the need to transmit patient data to a centralized location. This decentralization of data ensures privacy and confidentiality of patient information is maintained.
- MRI machines can vary in their hardware specifications and imaging protocols, which can lead to challenges in standardizing image reconstruction algorithms. However, FL accommodates this heterogeneity by allowing models to be trained collaboratively across different types of MRI machines, ensuring that the reconstruction algorithms are robust and adaptable to various configurations.

It is worth noting that FL models are predominantly supervised and have been developed under the end-to-end and unroll model frameworks, which have shown promising results in various applications.

### 3.4 Self-supervised learning

In contrast to supervised learning methods that necessitate fully sampled ground truth images, self-supervised models alleviate this requirement and are often trained using unrolling optimization techniques. The training framework of self-supervised algorithms does not mandate fully sampled ground truth images. This approach is particularly advantageous in scenarios where obtaining fully sampled data without distortions is challenging, such as myocardial perfusion with the patient’s involuntary movements, which cause motion artifacts (Haji-Valizadeh et al., 2018). Self-supervised methods draw samples from the undersampled pattern *Ω* provided in (3) to generate a new pattern *Λ* and *Θ*, where *Ω* = *Λ* ∪ *Θ* and *Θ* = *Ω* \ *Λ*. The former is used to train the DL model, while the latter is utilized to compute the loss (Feng et al., 2023; Heydari et al., 2024).

### 3.5 Assessment

In the previous sections, we covered three major DL-based CS-MRI training approaches: end-to-end, unroll optimization, and the DC layer. While these methods can be applied to both supervised and self-supervised frameworks, the unroll optimization and DC layer approaches are typically used for self-supervised training. For your convenience, we summarized the advantages and disadvantages of each approach in Table 8.

## 4 Evaluation metrics

### 4.1 Quantitative metrics

When testing CS-MRI models, the quality of reconstructed images is quantitatively assessed by comparing them with the ground truth. Retrospective undersampling of the k-space allows the reporting of indices to quantify the quality of the reconstructed images.

**Figure 9:**
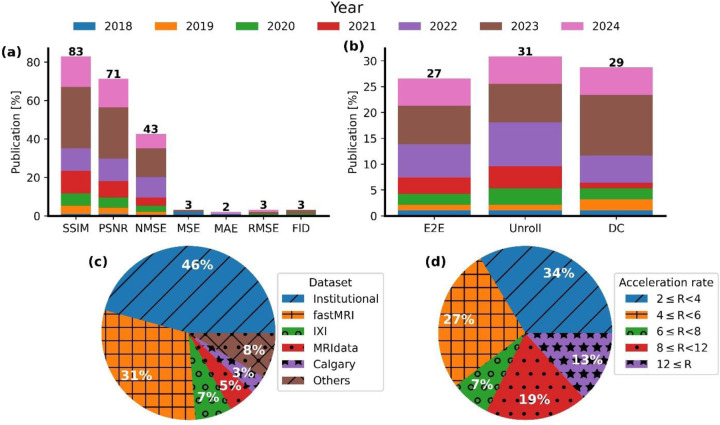
This graph illustrates (a) The metric used in different studies, (b) the training method used in different studies, (c) the dataset used for training used in the studies, and (d) the acceleration rate of the undersampling. Abbreviations: SSIM: structural similarity index; PSNR: peak signal-to-noise ratio; NMSE: normalized mean square error; NRMSE: normalized root mean square error; MSE: mean square error; MAE: mean absolute error; RMSE: root mean square error; and FID: Fréchet inception distance.

Most studies quantitatively compare predicted images with ground truth images. As indicated in Figure 9a, most of these studies use the structural similarity (SSIM) index or peak signal-to-noise ratio (PSNR) to compare reconstructed image $\hat{x}$ with ground truth image *x* as follows:

(5) $$\text{PSNR}(\hat{x},x)={\text{log}}_{10}\frac{{\text{L}}^{2}}{\frac{1}{\text{N}}{\left\|\hat{x}-x\right\|}_{2}^{2}},$$

where ∥⋅∥_2_ is the squared Euclidean distance, *N* is the number of images’ voxels, and *L* is the maximum voxel intensity of *x*. A higher PSNR indicates a better reconstruction. The logarithmic operator quantifies image quality that closely aligns with human perception (Safari et al., 2023a).

(6) $$\text{SSIM}(\hat{x},\text{x})=\frac{\left(2\mu_{\hat{x}}\mu_{\text{x}}+{\text{c}}_{1})(2\sigma_{\hat{x}x}+{\text{c}}_{2}\right)}{\left(\mu_{\hat{x}}^{2}+\mu_{\text{x}}^{2}+{\text{c}}_{1})(\sigma_{\hat{x}}^{2}+\sigma_{\text{x}}^{2}+c_{2}\right)},$$

where $\mu_{\hat{x}}$ and *μ*_*x*_ are the average voxel intensities in $\hat{x}$ and *x*, $\sigma_{\hat{x}}$ and *σ*_*x*_ are the variance, and $\sigma_{\hat{x}x}$ is the covariance between $\hat{x}$ and *x*. The constants *c*_1_ and *c*_2_ stabilizes the division, which usually are *c*_1_ = (*k*_1_*L*)^2^ and *c*_2_ = (*k*_2_*L*)^2^. SSIM ranges between −1 and 1, with the best similarity achieved by an SSIM equal to one.

The normalized mean square error (NMSE) has become more popular since 2022 to quantify the quality of reconstructed images. The NMSE is defined as

(7) $$\text{NMSE}(\hat{x},\text{x})=\frac{∥\hat{x}-\text{x}{∥}_{2}^{2}}{∥\text{x}{∥}_{2}^{2}},$$

Smaller NMSE values indicate better image reconstruction. However, it favors image smoothness rather than sharpness.

However, other metrics such as root mean square error, mean square error, mean absolute error, and Fréchet inception distance are rarely used, especially after a recommendation made in 2018 by Zbontar, Jure, *et al*. (Zbontar et al., 2018) (see Figure 8a).

Regarding the training methods, the Unroll models, including the Unroll optimization and DC layer, are the most commonly used, with a growing use of the DC layer since 2020, which is expected to continue this trend in the future. More details about these trends are illustrated in Figure 9b.

Our systematic review found that around 46% of studies used their own dataset, while fastMRI was the most frequently used public dataset. The majority of private datasets use single-coil images compared to the later’s raw multi-coil raw 2D *k*-space data. The fastMRI dataset consists of three imaging regions: the brain, pelvis, and knee regions. Less commonly used datasets include IXI, MRIdata, Calgary, and MICCAI challenges, with only around 7%, 5%, and 3.5% usage rates, respectively (see Figure 9c).

In addition, most studies reviewed by this study tested their proposed model using acceleration factor (R) 2 ≤ *R* < 6. The least simulated acceleration factor was *R* ≥ 12 and 6 ≤ *R* < 8 with 13.4% and 7.3% of usage, respectively. The percentage of acceleration usage is summarized in Figure 9d.

### 4.2 Clinical evaluations

The metrics presented in Section 4.1 quantify the quality of image reconstruction, but their results may not directly correlate with clinical outcomes. Several studies have been conducted to evaluate the clinical significance of CS-MRI using DL models. For example, a study found that DL-based CS-MRI and fully sampled MRI images showed no significant differences (p-values > 0.05) in the organ-based image quality of the liver, pancreas, spleen, and kidneys, number and measured diameter of the detected lesions while reducing the imaging time by more than 85% (Herrmann et al., 2023).

Similarly, another study showed that brain MRI images accelerated up to 4 × and 14 × had sufficient image quality for diagnostic and screening purposes, respectively (Radmanesh et al., 2022). A third study found that there was no statistical significance (*p*-value = 0.521) between the DL-based T2-FLAIR MRI image and standard T1c MRI images in the assessment of inflammatory knee synovitis (Feuerriegel et al., 2023). These studies are consistent with another conducted to compare the diagnostic performance of DL MRI and standard MRI images in detecting knee abnormalities (Johnson et al., 2023).

A recent study found that there is no significant difference in the overall quality of MRI images generated by DL and standard fully sampled images for various MRI sequences, including T2 and diffusion-weighted imaging, for patients with prostate cancer. The study also revealed that DL MRI and standard MR images identified a similar number of Prostate Imaging Reporting and Data System (PIRADS) ≥ 3 lesions. However, the imaging time was significantly reduced by about 3.7-fold with the use of DL MRI. This study’s findings suggest that DL MRI can be a viable alternative to standard MRI imaging for prostate cancer patients, as it can produce similar quality images in a significantly shorter acquisition time (Johnson et al., 2022).

## 5 Discussion

The rapid advancement of DL in the field of computer vision has led to a significant increase in the number of studies utilizing DL to solve CS-MRI, as depicted in Figure 7. In this study, we provide a comprehensive overview of DL models and training approaches, including the use of GAN and neural architecture such as U-net and vision transformers for an end-to-end approach. Recent studies have extensively used the unroll optimization and DC layers due to their advantages, allowing for the employment of smaller ConvNets. This, in turn, reduces the number of trainable parameters, minimizing the risk of overfitting. Moreover, our study also revealed that the majority of studies employ SSIM, PSNR, and NMSE metrics to measure the image quality of predicted images.

The objective of DL-based CS-MRI approaches is to decrease imaging time, thereby enhancing throughput and minimizing the chances of patient movement. Apart from this, other methods, such as super-resolution and image synthesis, can also be adopted to decrease imaging time. These approaches strive to enhance the resolution of images obtained from lower spatial resolution (Chang et al., 2024; Xie et al., 2022) and produce MRI images of high spatial resolution and signal-to-noise ratio from lower B_0_ (Eidex et al., 2023). These techniques can significantly improve MRI imaging with permanent magnets having low B_0_, ultimately increasing the image quality. Portable MRI scanners widely use permanent magnets, which substantially reduce maintenance costs and make them more suitable for low-income and middle-income countries. Although these techniques can reduce the need for stronger gradient magnets and minimize patients’ nerve stimulation, they were not included in this systematic review since they did not employ compressed sensing algorithms to train their models.

The coil sensitivity map, depicted in Figure 2b, is essential to consider the non-uniform sensitivity of the receiver coils. A precise sensitivity map is crucial for generating consistent and accurate MRI images and quantitative MRI maps across different hospitals. Most of the reviewed studies use the ESPIRiT algorithm (Uecker et al., 2014) provided by the Berkeley Advanced Reconstruction Toolbox (https://mrirecon.github.io/bart/) to pre-calculate the sensitivity map. However, this algorithm involves significant computation that may hinder its application in MRI-guided surgery and treatment, where rapid image reconstruction is required. We anticipate that DL-based CS-MRI techniques, which can simultaneously predict coil sensitivity maps and coil images (Feng et al., 2023; Sun et al., 2023; Z. Wang et al., 2024), will be explored further in the future, particularly for MRI-guided treatment methods such as MRI-guided adaptive radiation therapy. Furthermore, most CS-MRI methods require prior knowledge of the sampling pattern, which may not be available to the user in real clinical applications. Therefore, an approach that can handle deviations from the actual sampling mask or jointly predict the optimal sampling pattern and predict the images (Seo et al., 2022) is required.

In a recent systematic review by Hu, Mingzhe, *et al*., the use of language models in medical imaging was explored in detail (Hu et al., 2024). However, our comprehensive review has found that the utilization of foundational models in training DL-based CS-MRI is currently quite limited. We believe that these models could have a more significant impact on DL-based CS-MRI. By providing prior knowledge about the sampling pattern without explicitly specifying the sampling images, these models could be extremely helpful. Additionally, their prior inputs about the MRI sequence and imaging region could aid in training a model specific to that region and sequence, potentially improving the reconstruction of out-ofdistribution and out-of-region images.

### 6 Concluding remarks

The current DL-based CS-MRI models are usually trained by using 2D fastMRI datasets, limiting the spatial resolution for small lesion detection. With the advancement of MRI techniques, future research can significantly benefit from the availability of large 3D or 4D raw *k*-space datasets featuring abnormalities. Such datasets can enable the development of tailored 3D models, potentially allowing real-time tumor tracking during radiation therapy for patients with conditions like lung cancer. By incorporating high-dimensional datasets, it would be possible to report accurate clinical endpoints to enhance cancer prognosis.

## Figures and Tables

**Table 1.** Related review papers from the DL-based CS-MRI.

References	Year	Contributions and technical differences
(Yoon et al., 2023)	2023	This review focuses on accelerated musculoskeletal MRI. It does not discuss the statistics on the quantitative metrics and acceleration rates.
(Y. Chen et al., 2022)	2022	This review details DL algorithms and provided statistics on quantitative metrics. However, our comprehensive review, in addition to those statistics, provided detailed explanations about MRI imaging, such as k-space trajectories, the implications of different sampling patterns, and parallel imaging. Our comprehensive review also discussed the clinical applications of DL-based CS-MRI and provides insights into future directions.
(Bustin et al., 2020)	2020	The primary consideration is on DL-based CS-MRI for cardiac imaging. In addition to being wider in scope, our review discusses clinical applications of interest to imaging centers, provides relevant statistics, categorizes the DL method used, and lists related references.
(Xie and Li, 2022)	2022	A review about CS for medical applications. We focus on CS for MRI and categorize based on the study’s training method. In addition, our comprehensive review provides details about MRI acquisition and acceleration methods.

**Table 2:** The widely used regularization terms in deep CS-MRI are summarized.

Traditional	DL-based model	Regularizer	Reference
Dictionary learning	ADMS	$\|\alpha {\|}_{1}$	(Cao et al., 2020)
Field of expert	VN	$\sum_{i}\langle \Phi_{i}(K_{i}x),1\rangle$	(Hammernik et al., 2018)
pISTA-SENSE	plSTA-SENSE-ResNet	$\|\Psi x{\|}_{1}$	(Lu et al., 2020)
Total variation	-, RELAX	$\|\nabla x{\|}_{1}$	(F. Liu et al., 2021; Sun et al., 2017)
Sparse and low-rank model	ODLS	$\|ℋx{\|}_{*}$	(Z. Wang et al., 2022)

**Dictionary learning** learns a latent representation *α* of the input image *x* where the *ℓ*_1_ norm enforces it to be sparse.

**Field of expert** is composed of the convolution kernel *K*_*i*_ and Φ_*i*_, which they are learned from data.

**pISTA-SENSE** is a projected iterative soft-thresholding algorithm that solves (1) iteratively where the transform Ψ enforces the sparsity of the reconstructed image *x*.

**Total variation** enforces image smoothness by minimizing the image gradient variations.

**Sparse and low-rank model** minimize the nuclear norm ‖ℋ*x*‖_*_ where ℋ is the Hankel matrix.

**Table 3:** Overview of supervised end-to-end models to predict the fully sampled images.

DL Model	Dataset	Region	Modality	sample size	R	Ref.	Code^1^
GAN	fastMRI^2^ (Zbontar et al., 2018)	Knee	PDFS,PD	299,300	4,6	(Narnhofer et al., 2019)	No
GAN	IXI^3^ NAMIC^4^	Brain	T2,PD T1,T2	72 20	2,3,4	(Lyu et al., 2020)	No
ResNet	(Hammernik et al., 2018)	knee	PD	15	5,7,9	(Lu et al., 2020)	Yes
U-net	Institutional^5^	Knee	T1*ρ*-map, T2-map	10	2,4,5,6	(Li et al., 2023)	No
GAN	MICCAI 2013^6^ ASC^7^ fastMRI	Brain Cardiac Knee	T1 T1c PD	150 100 96	2,3.3,10	(Gao et al., 2023)	No
Y-net	Own	Brain	T1,T2	37	2,3,4,5,6,8	(Do et al., 2020)	No
U-net	Own	Knee	T1	10	3,4,5	(Ayde et al., 2022)	No
SwinGAN	IXI MICCAI 2013 MRNet	Brain Brain Knee	T1 NS^8^ NS	573 2480^9^ 2480^9^	20,30,50	(Zhao et al., 2023)	Yes
Dense-Unet	MICCAI 2016	Brain	T2	5	2,4,8	(Xiang et al., 2018)	No
U-net	Institutional	Cardiac	bSSFP,GRE	80	NS	(J. Wang et al., 2024)	No
GAN (Pix2pix)	HCP Institutional	Brain	T1,T2 T2-map	20 NS	8	(Meng et al., 2021)	No
GAN	Institutional	Cardiac^10^	DTI	30	2.6	(Liu et al., 2023)	No
DeepADC-Net	Institutional	Animal study	DWI	183	4,8	(Li et al., 2024)	Yes
NPB-REC	fastMRI	Brain, Knee	NS	5847,1167	4,8,12	(Khawaled and Freiman, 2024)	Yes
SCU-Net	Institutional	Brain	T1,T2,T2FLAIR	180	1.78,2.39, 2.91, 3.33,3.64, 4, 4.27,4.44, 4.71	(Jin and Xiang, 2023)	No
DuDReTL U-net	Uk Biobank^11^	Cardiac	NS	9032	20	(Hong et al., 2023)	Yes
iRAKI	fastMRI Institutional	Brain	T1,T2,T1c,T2FLAIR T1,T2,T1FS	10 3	4,5	(Dawood et al., 2023)	Yes
MA-RECON	fastMRI MRIdata^12^	Brain Knee	T2FLAIR PD	1000^9^ 41872^9^	4,8	(Avidan and Freiman, 2024)	Yes
Diffusion model	fastMRI	Knee	PD	973	10,12	(Cao et al., 2024)	Yes

1 Whether the original implementation is publicly available,

2 https://fastmri.med.nyu.edu/,

3 https://brain-development.org/ixi-dataset/,

4 https://insight-journal.org/,

5 The study used its Institutional dataset,

6 MICCAI 2013 grand challenge dataset: https://wiki.cancerimagingarchive.net/display/Public/NCI-MICCAI+2013+Grand+Challenges+in+Image+Segmentation,

7 Atrial Segmentation Challenge: https://www.cardiacatlas.org/atriaseg2018-challenge/,

8 Information not available from the publication,

9 Number of image slices was specified,

10 *Ex-vivo* study

11 https://www.ukbiobank.ac.uk/

12 https://www.mridata.org/

**Table 4:** Overview of supervised unroll optimization to predict the fully sampled images.

DL Model	Dataset	Region	modality	sample size	R	Reference	Code
ConvNet	IXI fastMRI	Brain Knee	T1,T2 PD	50 1025	2.5, 3.3, 5	(R. Liu et al., 2020)	Yes
ConvNet	Institutional fastMRI	Brain Knee	T1 PD, PDFS	5 60	4,5,6.7	(D. Guo et al., 2023)	No
RG-Net	Institutional	Brain	T1*ρ*	8	17	(Huang et al., 2017)	No
ConvNet	Institutional	Brain	SWI	117	5,8	(Duan et al., 2022)	No
ConvNet	HCP (Van Essen et al., 2013)	Brain	T1w,T2w	1200	NS^1^	(Zufiria et al., 2022)	No
ConvNet	Institutional	Cardiac, Abdominal	NS	20,16	3,4,5	(Zhou et al., 2019)	No
RecurrentVarNet	fastMRI fastMRI Calgary-Campinas (Beauferris et al., 2022)	Brain Knee Brain	T1c, T1, T2,T2- FLAIR PD T1w	5846 1172 67	2,4,6	(Yiase mis et al., 2024)	No
ConvNet	(Hammerni k et al., 2018) Institutional	Knee Brain	PD T1	20 8	3,4,6	(Hamm ernik et al., 2018)	No
UFLoss	fastMRI MRIdata	Knee Knee	PDFS, PD PDFS, PD	380 20	5 8	(K. Wang et al., 2022)	Yes
DFSN	SRI24 (Rohlfing et al., 2010) MRBrainS13(Mendrik et al., 2015) NeoBrainS12 (Išgum et al., 2015)	Brain	PD,T1,T2 T1,T2- FLAIR T1,T2	36 20 175	5, 10	(Sun et al., 2019)	No
ConvNet	Institutional	Brain	T1map	3	8,12,18, 36	(Slavko va et al., 2023)	Yes
JDL	Institutional	Brain	T1,T2,PD,T2 FLAIR	8	4,8	(Ryu et al., 2021)	Yes
pFISTA-DR	Institutional	Brain	T1,PD	200	5,7,10	(Qu et al., 2024)	No
U-net	fastMRI	Knee	PD, PDFS	20	4,6	(Qiao et al., 2023)	Yes
ConvNet	fastMRI Institutional	Brain	T1,T2,T2-FLAIR	120 3	1.8,2.5,3.5,4	(Prama nik et al., 2023)	No
ConvNet	NAMIC^2^ MRBrainS^3^	Brain Brain	T1,T2 T2,T2-FLAIR	5 7	8	(X. Liu et al., 2021)	No
Dictionary learning	Institutional	Cardiac	-	19	2,4,8	(Kofler et al., 2023)	Yes
PD-PCG-Net	fastMRI	Knee	PD, PDFS	484,489	4	(Kim and Chung, 2022)	No
RUN-UP	Institutional	Brain, Breast	DTI, DWI	14,6	100^4^	(Y. Hu et al., 2021)	No
ConvNet	Institutional	Cardiac	NS	22	10,12,14	(Sandin o et al., 2021)	No
CEST-VN	Institutional	Brain	CEST	54	3,4,5	(Xu et al., 2024)	Yes
Diffusion model	GLIS-RT	Brain	T1c	230	1.25,1.66,2.5,5	(Safari et al., 2024)	No

1 It is not specified in the manuscript

2 http://hdl.handle.net/1926/1687

3 https://mrbrains18.isi.uu.nl/

**Table 5:** Overview of supervised models that used the data consistency to predict the fully sampled images.

DL Model	Dataset	Region	Modality	sample size	R	Ref.	Code
KTMR	Institutional	Brain	T1c, MRA	17	2,2.5,3.3,5,10	(Wu et al., 2023)	Yes
DCT-net	fastMRI Calgary	Brain Knee	T1 PD	973 25	4,8	(B. Wang et al., 2024)	No
CTFNet	Institutional	Cardiac	bSSFP	48	8,16,24	(Qin et al., 2021)	Yes
EDAEPRec	Institutional	Brain	T2	7	33,4,5,6.7,10	(Q. Liu et al., 2020)	No
GFN	Institutional	Brain	T1MPRAGE,T2FLAIR,TOF	30,50,80	3.3,5,10	(Dai et al., 2023)	No
PC-RNN	fastMRI	Knee Brain	PD, PDFS T1,T2	973 4469	4,6	(E. Z. Chen et al., 2022)	Yes
ResNet Unet	Institutional	Knee	T1	360	6	(Wu et al., 2019)	No
CNF	fastMRI	Knee,Brain	PD, T2	20,8	4	(Wen et al., 2023)	Yes
stDLNN	Institutional	Abdomen	GRE	8	4,8,16,25	(Wang et al., 2023)	Yes
MODEST	Institutional	Cardiac		28	3.7,7.4,14.8	(Terpstra et al., 2023)	Yes
Deepcomplex MRI	Institutional (Hammernik et al., 2018)	Brain Knee	T1,T2,PD PD	22 20	4,5,10	(Wang et al., 2020)	No
AdaDiff	fastMRI IXI	Brain	T1, T2, PD T1,T2,T2-FLAIR	66 420	4,8	(Güngör et al., 2023)	Yes
McSTRA	fastMRI	Knee	PD, PDFS	584,588	4,6,8,10,12	(Ekanaya ke et al., 2024)	No
DC-RSN	Cardiac Kirby Calgary MRBrain Hammerni ck	Cardiac, Brain, Brain, Brain, Knee	Cardiac, T1-MPRAGE T1 T2-FLAIR PDF, PD, T1, T2, T2FS	200 42 45 7 25	4, 5	(Muruges an et al., 2021)	No

**Table 6.** Overview of supervised models that were trained with federated learning.

DL Model	Training framework	Dataset	sample size	Ref.	Code
FL-MRCM	End-to-End	fastMRI, HPKS(Jiang et al., 2019) IXI, BraTS(Menze et al., 2014)	3443 144 434 494	(Guo et al., 2021b)	Yes
FedMRI	End-to-End	fastMRI BraTS SMS (Feng et al., 2021) uMR (Feng et al., 2021)	2134 385 155 50	(Feng et al., 2022)	Yes
ACM-FedMRI	DC guided	fastMRI BraTS IXI Institutional	3443 494 526 150	(Lyu et al., 2023)	No
FedGIMP	DC guided	fastMRI BraTS IXI Institutional	51 55 55 10	(Elmas et al., 2022)	Yes
Unrolled FedLrn	Unroll	fastMRI, MRIdata	NS	(B. R. Levac et al., 2023)	Yes

**Table 7:** Overview of self-supervised models to predict the fully sampled images.

DL Model	Dataset	Region	modality	sample size	R	Ref.	Code
DC-SiamNet	IXI MRINet	Brain Knee	T1, T2, PD PD	473 1250	4, 5	(Yan et al., 2023)	No
Noise2Recon	MRIdata fastMRI	Knee Brain	PDFS T2,T2-FLAIR,T1,T1c	19 603	12,16	(Desai et al., 2023)	Yes
DDSS	HCP	Brain	T1	505	2,4	(Zhou et al., 2022)	No
SSDU	InstitutionalfastMRI fastMRI	Brain Knee Brain	T1MPRAGE PD,PDFS T2	19 35,25 10	4,6,8	(Yaman et al., 2020)	Yes
Multi-mask SSDU	Institutional MRIDdata	Brain Knee	T1MPRAGE PD	9 20	8,12	(Yaman et al., 2022)	No
RELAX	Institutional	Brain, Knee	T1 and T2 maps	21	5	(F. Liu et al., 2021)	No
Joint MAPLE	Institutional	Brain	T1 and T2* maps	2	16,25	(Heydari et al., 2024)	Yes

**Table 8:** A bullet list of the pros and cons of each training framework is provided.

DL method	Pros	Cons
End-to-end	Free from MRI data acquisition method Easy to employ models proposed for image synthesis and segmentation	Requires a big dataset Likely to add unwanted image structure, specifically for higher acceleration rate Limited generalization to out-ofdistribution data
Unroll optimization	Requires smaller datasets compared with the end-to-end method More likely to generalize better Performs well using a ConvNet with small number of trainable parameters	Iterative method Requires image acquisition knowledge Requires undersampling pattern in inference time
DC Layer	Requires smaller dataset than the end-to-end method Provides closed form equation Easy to implement	Requires undersampling pattern in inference time

## Abbreviations:

bSSFP: Balanced steady-state free precession
CS: compressed sensing
DC: Data consistency
DL: Deep learning
DL-based CS-MRI: Deep learning-based compressed sensing-magnetic resonance imaging
DTI: Diffusion-tensor imaging
DWI: Diffusion-weighted imaging
E2E: End-to-end
FID: Fréchet inception distance
FL: Federated learning
FLAIR: Fluid-attenuated inversion recovery
GRE: Gradient echo
MAE: Mean absolute error
MRA: Magnetic resonance angiography
MRI: Magnetic resonance imaging
MSE: Mean square error
NMSE: Normalized mean square error
PD: Proton density
PDFS: Proton density fat-suppression
PSNR: peak signal-to-noise ratio
R: Acceleration rate
RMSE: Root mean square error
RSS: Root sum of squares
T1c: T1-w post-contrast MRI
T1-MPRAGE: T1w magnetization-prepared rapid gradient-echo
SSIM: Structural similarity index
SWI: Susceptibility-weighted imaging

## References

[R1] Aggarwal H.K., Mani M.P., Jacob M., 2018. MoDL: Model-based deep learning architecture for inverse problems. IEEE transactions on medical imaging 38, 394–405. 30106719 10.1109/TMI.2018.2865356 PMC6760673

[R2] Avidan N., Freiman M., 2024. MA-RECON: Mask-aware deep-neural-network for robust fast MRI k-space interpolation. Computer Methods and Programs in Biomedicine 244, 107942. 38039921 10.1016/j.cmpb.2023.107942

[R3] Ayde R., Senft T., Salameh N., Sarracanie M., 2022. Deep learning for fast low-field MRI acquisitions. Scientific reports 12, 11394. 35794175 10.1038/s41598-022-14039-7 PMC9259619

[R4] Balakrishnan G., Zhao A., Sabuncu M.R., Guttag J., Dalca A.V., 2019. Voxelmorph: a learning framework for deformable medical image registration. IEEE transactions on medical imaging 38, 1788–1800. 10.1109/TMI.2019.2897538 30716034

[R5] Beauferris Y., Teuwen J., Karkalousos D., Moriakov N., Caan M., Yiasemis G., Rodrigues L., Lopes A., Pedrini H., Rittner L., others, 2022. Multi-coil mri reconstruction challenge—assessing brain mri reconstruction models and their generalizability to varying coil configurations. Frontiers in Neuroscience 16, 919186. 35873808 10.3389/fnins.2022.919186 PMC9298878

[R6] Bustin A., Fuin N., Botnar R.M., Prieto C., 2020. From Compressed-Sensing to Artificial Intelligence-Based Cardiac MRI Reconstruction. Front. Cardiovasc. Med. 7, 17. 10.3389/fcvm.2020.00017 32158767 PMC7051921

[R7] Cao C., Cui Z.-X., Wang Y., Liu S., Chen T., Zheng H., Liang D., Zhu Y., 2024. High-Frequency Space Diffusion Model for Accelerated MRI. IEEE Transactions on Medical Imaging. 10.1109/TMI.2024.3351702 38194398

[R8] Cao J., Liu S., Liu H., Lu H., 2020. CS-MRI reconstruction based on analysis dictionary learning and manifold structure regularization. Neural Networks 123, 217–233. 31884182 10.1016/j.neunet.2019.12.010

[R9] Chan S.H., 2024. Tutorial on Diffusion Models for Imaging and Vision. arXiv preprint arXiv:2403.18103.

[R10] Chang C.-W., Peng J., Safari M., Salari E., Pan S., Roper J., Qiu R.L., Gao Y., Shu H.-K., Mao H., others, 2024. High-resolution MRI synthesis using a data-driven framework with denoising diffusion probabilistic modeling. Physics in Medicine & Biology 69, 045001. 10.1088/1361-6560/ad209c PMC10839468 38241726

[R11] Chen E.Z., Wang P., Chen X., Chen T., Sun S., 2022. Pyramid convolutional RNN for MRI image reconstruction. IEEE Transactions on Medical Imaging 41, 2033–2047. 35192462 10.1109/TMI.2022.3153849

[R12] Chen Y., Schonlieb C.-B., Lio P., Leiner T., Dragotti P.L., Wang G., Rueckert D., Firmin D., Yang G., 2022. AI-Based Reconstruction for Fast MRI—A Systematic Review and Meta-Analysis. Proc. IEEE 110, 224–245. 10.1109/JPROC.2022.3141367

[R13] Cheng J., Cui Z.-X., Huang W., Ke Z., Ying L., Wang H., Zhu Y., Liang D., 2021. Learning data consistency and its application to dynamic MR imaging. IEEE Transactions on Medical Imaging 40, 3140–3153. 34252025 10.1109/TMI.2021.3096232

[R14] Chung H., Ye J.C., 2022. Score-based diffusion models for accelerated MRI. Medical image analysis 80, 102479. 35696876 10.1016/j.media.2022.102479

[R15] Dai Y., Wang C., Wang H., 2023. Deep compressed sensing MRI via a gradient-enhanced fusion model. Medical Physics 50, 1390–1405. 36695158 10.1002/mp.16164

[R16] Dawood P., Breuer F., Stebani J., Burd P., Homolya I., Oberberger J., Jakob P.M., Blaimer M., 2023. Iterative training of robust k-space interpolation networks for improved image reconstruction with limited scan specific training samples. Magnetic Resonance in Medicine 89, 812–827. 36226661 10.1002/mrm.29482

[R17] Desai A.D., Ozturkler B.M., Sandino C.M., Boutin R., Willis M., Vasanawala S., Hargreaves B.A., Ré C., Pauly J.M., Chaudhari A.S., 2023. Noise2Recon: Enabling SNR-robust MRI reconstruction with semi-supervised and self-supervised learning. Magnetic Resonance in Medicine 90, 2052–2070. 37427449 10.1002/mrm.29759

[R18] Deshmane A., Gulani V., Griswold M.A., Seiberlich N., 2012. Parallel MR imaging. Magnetic Resonance Imaging 36, 55–72. 10.1002/jmri.23639 PMC4459721 22696125

[R19] Do W.-J., Seo S., Han Y., Ye J.C., Choi S.H., Park S.-H., 2020. Reconstruction of multicontrast MR images through deep learning. Medical physics 47, 983–997. 31889314 10.1002/mp.14006

[R20] Dong X., Lei Y., Wang T., Thomas M., Tang L., Curran W.J., Liu T., Yang X., 2019. Automatic multiorgan segmentation in thorax CT images using U-net-GAN. Medical physics 46, 2157–2168. 30810231 10.1002/mp.13458 PMC6510589

[R21] Donoho D.L., 2006. For most large underdetermined systems of linear equations, the minimal ℓ1 solution is also the sparsest solution. Communications on pure and applied mathematics 59, 797–829.

[R22] Dosovitskiy A., Beyer L., Kolesnikov A., Weissenborn D., Zhai X., Unterthiner T., Dehghani M., Minderer M., Heigold G., Gelly S., others, 2020. An image is worth 16x16 words: Transformers for image recognition at scale. arXiv preprint arXiv:2010.11929.

[R23] Duan C., Xiong Y., Cheng K., Xiao S., Lyu J., Wang C., Bian X., Zhang J., Zhang D., Chen L., others, 2022. Accelerating susceptibility-weighted imaging with deep learning by complex-valued convolutional neural network (ComplexNet): validation in clinical brain imaging. European Radiology 32, 5679–5687. 35182203 10.1007/s00330-022-08638-1

[R24] Eidex Z., Wang J., Safari M., Elder E., Wynne J., Wang T., Shu H.-K., Mao H., Yang X., 2023. High-resolution 3T to 7T MRI Synthesis with a Hybrid CNN-Transformer Model. arXiv preprint arXiv:2311.15044. 10.1002/mp.17079 PMC11650373 38630982

[R25] Ekanayake M., Pawar K., Harandi M., Egan G., Chen Z., 2024. McSTRA: A multi-branch cascaded swin transformer for point spread function-guided robust MRI reconstruction. Computers in Biology and Medicine 168, 107775. 38061154 10.1016/j.compbiomed.2023.107775

[R26] Elmas G., Dar S.U., Korkmaz Y., Ceyani E., Susam B., Ozbey M., Avestimehr S., Çukur T., 2022. Federated learning of generative image priors for MRI reconstruction. IEEE Transactions on Medical Imaging. 10.1109/TMI.2022.3220757 36350868

[R27] Fang J., Lin H., Chen X., Zeng K., 2022. A hybrid network of cnn and transformer for lightweight image super-resolution, in: Proceedings of the IEEE/CVF Conference on Computer Vision and Pattern Recognition. pp. 1103–1112.

[R28] Feng C.-M., Fu H., Yuan S., Xu Y., 2021. Multi-contrast MRI super-resolution via a multi-stage integration network, in: Medical Image Computing and Computer Assisted Intervention–MICCAI 2021: 24th International Conference, Strasbourg, France, September 27–October 1, 2021, Proceedings, Part VI 24. Springer, pp. 140–149.

[R29] Feng C.-M., Yan Y., Wang S., Xu Y., Shao L., Fu H., 2022. Specificity-preserving federated learning for MR image reconstruction. IEEE Transactions on Medical Imaging. 10.1109/TMI.2022.3202106 36018876

[R30] Feng R., Wu Q., Feng J., She H., Liu C., Zhang Y., Wei H., 2023. IMJENSE: scan-specific implicit representation for joint coil sensitivity and image estimation in parallel MRI. IEEE Transactions on Medical Imaging. 10.1109/TMI.2023.3342156 38090839

[R31] Feuerriegel G.C., Goller S.S., von Deuster C., Sutter R., 2023. Inflammatory Knee Synovitis: Evaluation of an Accelerated FLAIR Sequence Compared With Standard Contrast-Enhanced Imaging. Investigative Radiology 10–1097. 10.1097/RLI.0000000000001065 38329824

[R32] Gao Z., Guo Y., Zhang J., Zeng T., Yang G., 2023. Hierarchical perception adversarial learning framework for compressed sensing MRI. IEEE Transactions on Medical Imaging. 10.1109/TMI.2023.3240862 37022266

[R33] Geng C., Jiang M., Fang X., Li Y., Jin G., Chen A., Liu F., 2023. HFIST-Net: High-throughput fast iterative shrinkage thresholding network for accelerating MR image reconstruction. Computer Methods and Programs in Biomedicine 232, 107440. 36881983 10.1016/j.cmpb.2023.107440

[R34] Goodfellow I., Pouget-Abadie J., Mirza M., Xu B., Warde-Farley D., Ozair S., Courville A., Bengio Y., 2020. Generative adversarial networks. Communications of the ACM 63, 139–144.

[R35] Güngör A., Dar S.U., Öztürk Ş., Korkmaz Y., Bedel H.A., Elmas G., Ozbey M., Çukur T., 2023. Adaptive diffusion priors for accelerated MRI reconstruction. Medical Image Analysis 88, 102872. 37384951 10.1016/j.media.2023.102872

[R36] Guo D., Zeng G., Fu H., Wang Z., Yang Y., Qu X., 2023. A Joint Group Sparsity-based deep learning for multi-contrast MRI reconstruction. Journal of Magnetic Resonance 346, 107354. 36527935 10.1016/j.jmr.2022.107354

[R37] Guo P., Mei Y., Zhou J., Jiang S., Patel V.M., 2023. ReconFormer: Accelerated MRI reconstruction using recurrent transformer. IEEE transactions on medical imaging. 10.1109/TMI.2023.3314747 PMC10764001 37703139

[R38] Guo P., Valanarasu J.M.J., Wang P., Zhou J., Jiang S., Patel V.M., 2021a. Over-and-under complete convolutional rnn for mri reconstruction, in: Medical Image Computing and Computer Assisted Intervention–MICCAI 2021: 24th International Conference, Strasbourg, France, September 27–October 1, 2021, Proceedings, Part VI 24. Springer, pp. 13–23. 10.1007/978-3-030-87231-1_2 PMC8517933 34661201

[R39] Guo P., Wang P., Zhou J., Jiang S., Patel V.M., 2021b. Multi-Institutional Collaborations for Improving Deep Learning-Based Magnetic Resonance Image Reconstruction Using Federated Learning, in: Proceedings of the IEEE/CVF Conference on Computer Vision and Pattern Recognition (CVPR). pp. 2423–2432. 10.1109/cvpr46437.2021.00245 PMC9017654 35444379

[R40] Haji-Valizadeh H., Rahsepar A.A., Collins J.D., Bassett E., Isakova T., Block T., Adluru G., DiBella E.V., Lee D.C., Carr J.C., others, 2018. Validation of highly accelerated real-time cardiac cine MRI with radial k-space sampling and compressed sensing in patients at 1.5 T and 3T. Magnetic resonance in medicine 79, 2745–2751. 28921631 10.1002/mrm.26918 PMC5821536

[R41] Haldar J.P., Hernando D., Liang Z.-P., 2010. Compressed-sensing MRI with random encoding. IEEE transactions on Medical Imaging 30, 893–903. 20937579 10.1109/TMI.2010.2085084 PMC3271122

[R42] Hammernik K., Klatzer T., Kobler E., Recht M.P., Sodickson D.K., Pock T., Knoll F., 2018. Learning a variational network for reconstruction of accelerated MRI data. Magnetic resonance in medicine 79, 3055–3071. 29115689 10.1002/mrm.26977 PMC5902683

[R43] Han X., 2017. MR-based synthetic CT generation using a deep convolutional neural network method. Medical physics 44, 1408–1419. 28192624 10.1002/mp.12155

[R44] He K., Zhang X., Ren S., Sun J., 2016. Deep residual learning for image recognition, in: Proceedings of the IEEE Conference on Computer Vision and Pattern Recognition. pp. 770–778.

[R45] Herrmann J., Wessling D., Nickel D., Arberet S., Almansour H., Afat C., Afat S., Gassenmaier S., Othman A.E., 2023. Comprehensive clinical evaluation of a deep learning-accelerated, single-breath-hold abdominal HASTE at 1.5 T and 3 T. Academic Radiology 30, 93–102. 35469719 10.1016/j.acra.2022.03.018

[R46] Heydari A., Ahmadi A., Kim T.H., Bilgic B., 2024. Joint MAPLE: Accelerated joint T1 and T 2* T _2^ * mapping with scan-specific self-supervised networks. Magnetic Resonance in Medicine. 10.1002/mrm.29989 PMC11007829 38181183

[R47] Hong G.Q., Wei Y.T., Morley W.A., Wan M., Mertens A.J., Su Y., Cheng H.-L.M., 2023. Dual-domain accelerated MRI reconstruction using transformers with learning-based undersampling. Computerized Medical Imaging and Graphics 106, 102206. 36857952 10.1016/j.compmedimag.2023.102206

[R48] Hricak H., Abdel-Wahab M., Atun R., Lette M.M., Paez D., Brink J.A., Donoso-Bach L., Frija G., Hierath M., Holmberg O., others, 2021. Medical imaging and nuclear medicine: a Lancet Oncology Commission. The Lancet Oncology 22, e136–e172. 33676609 10.1016/S1470-2045(20)30751-8 PMC8444235

[R49] Hu C., Li C., Wang H., Liu Q., Zheng H., Wang S., 2021. Self-supervised learning for mri reconstruction with a parallel network training framework, in: Medical Image Computing and Computer Assisted Intervention–MICCAI 2021: 24th International Conference, Strasbourg, France, September 27–October 1, 2021, Proceedings, Part VI 24. Springer, pp. 382–391.

[R50] Hu M., Qian J.Y., Pan S., Li Y., Qiu R.L., Yang X., 2024. Advancing medical imaging with language models: featuring a spotlight on ChatGPT. Physics in Medicine and Biology. 10.1088/1361-6560/ad387d PMC11075180 38537293

[R51] Hu Y., Xu Y., Tian Q., Chen F., Shi X., Moran C.J., Daniel B.L., Hargreaves B.A., 2021. RUN-UP: Accelerated multishot diffusion-weighted MRI reconstruction using an unrolled network with U-Net as priors. Magnetic resonance in medicine 85, 709–720. 32783339 10.1002/mrm.28446 PMC8095163

[R52] Huang G., Liu Z., Van Der Maaten L., Weinberger K.Q., 2017. Densely connected convolutional networks, in: Proceedings of the IEEE Conference on Computer Vision and Pattern Recognition. pp. 4700–4708.

[R53] Huang J., Fang Y., Wu Y., Wu H., Gao Z., Li Y., Del Ser J., Xia J., Yang G., 2022. Swin transformer for fast MRI. Neurocomputing 493, 281–304.

[R54] Hyun C.M., Kim H.P., Lee S.M., Lee S., Seo J.K., 2018. Deep learning for undersampled MRI reconstruction. Physics in Medicine & Biology 63, 135007. 29787383 10.1088/1361-6560/aac71a

[R55] Išgum I., Benders M.J., Avants B., Cardoso M.J., Counsell S.J., Gomez E.F., Gui L., H\Huppi P.S., Kersbergen K.J., Makropoulos A., others, 2015. Evaluation of automatic neonatal brain segmentation algorithms: the NeoBrainS12 challenge. Medical image analysis 20, 135–151. 25487610 10.1016/j.media.2014.11.001

[R56] Isola P., Zhu J.-Y., Zhou T., Efros A.A., 2017. Image-to-image translation with conditional adversarial networks, in: Proceedings of the IEEE Conference on Computer Vision and Pattern Recognition. pp. 1125–1134.

[R57] Jiang S., Eberhart C.G., Lim M., Heo H.-Y., Zhang Y., Blair L., Wen Z., Holdhoff M., Lin D., Huang P., others, 2019. Identifying recurrent malignant glioma after treatment using amide proton transfer-weighted MR imaging: a validation study with image-guided stereotactic biopsy. Clinical Cancer Research 25, 552–561. 30366937 10.1158/1078-0432.CCR-18-1233 PMC6335169

[R58] Jin Z., Xiang Q.-S., 2023. Improving accelerated MRI by deep learning with sparsified complex data. Magnetic Resonance in Medicine 89, 1825–1838. 36480017 10.1002/mrm.29556

[R59] Johnson P.M., Lin D.J., Zbontar J., Zitnick C.L., Sriram A., Muckley M., Babb J.S., Kline M., Ciavarra G., Alaia E., others, 2023. Deep learning reconstruction enables prospectively accelerated clinical knee MRI. Radiology 307, e220425. 36648347 10.1148/radiol.220425 PMC10102623

[R60] Johnson P.M., Tong A., Donthireddy A., Melamud K., Petrocelli R., Smereka P., Qian K., Keerthivasan M.B., Chandarana H., Knoll F., 2022. Deep learning reconstruction enables highly accelerated biparametric MR imaging of the prostate. Journal of Magnetic Resonance Imaging 56, 184–195. 34877735 10.1002/jmri.28024 PMC9170839

[R61] Karras T., Laine S., Aila T., 2019. A style-based generator architecture for generative adversarial networks, in: Proceedings of the IEEE/CVF Conference on Computer Vision and Pattern Recognition. pp. 4401–4410. 10.1109/TPAMI.2020.2970919 32012000

[R62] Khawaled S., Freiman M., 2024. NPB-REC: A non-parametric Bayesian deep-learning approach for undersampled MRI reconstruction with uncertainty estimation. Artificial Intelligence in Medicine 102798. 38462289 10.1016/j.artmed.2024.102798

[R63] Kim M., Chung W., 2022. A cascade of preconditioned conjugate gradient networks for accelerated magnetic resonance imaging. Computer Methods and Programs in Biomedicine 225, 107090. 36067702 10.1016/j.cmpb.2022.107090

[R64] Kofler A., Pali M.-C., Schaeffter T., Kolbitsch C., 2023. Deep supervised dictionary learning by algorithm unrolling—Application to fast 2D dynamic MR image reconstruction. Medical Physics 50, 2939–2960. 36565150 10.1002/mp.16182

[R65] Korkmaz Y., Cukur T., Patel V.M., 2023. Self-supervised MRI reconstruction with unrolled diffusion models, in: International Conference on Medical Image Computing and Computer-Assisted Intervention. Springer, pp. 491–501.

[R66] Krizhevsky A., Sutskever I., Hinton G.E., 2012. Imagenet classification with deep convolutional neural networks. Advances in neural information processing systems 25.

[R67] Larkman D.J., Nunes R.G., 2007. Parallel magnetic resonance imaging. Phys. Med. Biol. 52, R15–R55. 10.1088/0031-9155/52/7/R01 17374908

[R68] Lauterbur P.C., 1973. Image Formation by Induced Local Interactions: Examples Employing Nuclear Magnetic Resonance. Nature 242, 190–191. 10.1038/242190a0 2663289

[R69] LeCun Y., Bengio Y., others, 1995. Convolutional networks for images, speech, and time series. The handbook of brain theory and neural networks 3361, 1995.

[R70] Levac B., Jalal A., Tamir J.I., 2023. Accelerated motion correction for MRI using score-based generative models, in: 2023 IEEE 20th International Symposium on Biomedical Imaging (ISBI). IEEE, pp. 1–5.

[R71] Levac B.R., Arvinte M., Tamir J.I., 2023. Federated end-to-end unrolled models for magnetic resonance image reconstruction. Bioengineering 10, 364. 36978755 10.3390/bioengineering10030364 PMC10045102

[R72] Li H., Xu Z., Taylor G., Studer C., Goldstein T., 2018. Visualizing the loss landscape of neural nets. Advances in neural information processing systems 31.

[R73] Li H., Yang M., Kim J.H., Zhang C., Liu R., Huang P., Liang D., Zhang X., Li X., Ying L., 2023. SuperMAP: Deep ultrafast MR relaxometry with joint spatiotemporal undersampling. Magnetic resonance in medicine 89, 64–76. 36128884 10.1002/mrm.29411 PMC9617769

[R74] Li T., Sahu A.K., Zaheer M., Sanjabi M., Talwalkar A., Smith V., 2020. Federated optimization in heterogeneous networks. Proceedings of Machine learning and systems 2, 429–450.

[R75] Li Y., Joaquim M.R., Pickup S., Song H.K., Zhou R., Fan Y., 2024. Learning ADC maps from accelerated radial k-space diffusion-weighted MRI in mice using a deep CNN-transformer model. Magnetic Resonance in Medicine 91, 105–117. 37598398 10.1002/mrm.29833 PMC10691280

[R76] Liu F., Kijowski R., El Fakhri G., Feng L., 2021. Magnetic resonance parameter mapping using model-guided self-supervised deep learning. Magnetic resonance in medicine 85, 3211–3226. 33464652 10.1002/mrm.28659 PMC9185837

[R77] Liu Q., Yang Q., Cheng H., Wang S., Zhang M., Liang D., 2020. Highly undersampled magnetic resonance imaging reconstruction using autoencoding priors. Magnetic resonance in medicine 83, 322–336. 31429993 10.1002/mrm.27921

[R78] Liu R., Zhang Y., Cheng S., Luo Z., Fan X., 2020. A deep framework assembling principled modules for CS-MRI: unrolling perspective, convergence behaviors, and practical modeling. IEEE Transactions on Medical Imaging 39, 4150–4163. 32746155 10.1109/TMI.2020.3014193

[R79] Liu S., Li H., Liu Y., Cheng G., Yang G., Wang H., Zheng H., Liang D., Zhu Y., 2022. Highly accelerated MR parametric mapping by undersampling the k-space and reducing the contrast number simultaneously with deep learning. Physics in Medicine & Biology 67, 185004. 10.1088/1361-6560/ac8c81 36001990

[R80] Liu S., Liu Y., Xu X., Chen R., Liang D., Jin Q., Liu H., Chen G., Zhu Y., 2023. Accelerated cardiac diffusion tensor imaging using deep neural network. Physics in Medicine & Biology 68, 025008. 10.1088/1361-6560/acaa86 36595239

[R81] Liu X., Wang J., Sun H., Chandra S.S., Crozier S., Liu F., 2021. On the regularization of feature fusion and mapping for fast MR multi-contrast imaging via iterative networks. Magnetic resonance imaging 77, 159–168. 33400936 10.1016/j.mri.2020.12.019

[R82] Liu Y., Leong A.T., Zhao Y., Xiao L., Mak H.K., Tsang A.C.O., Lau G.K., Leung G.K., Wu E.X., 2021. A low-cost and shielding-free ultra-low-field brain MRI scanner. Nature communications 12, 7238. 10.1038/s41467-021-27317-1 PMC8671508 34907181

[R83] Liu Z., Lin Y., Cao Y., Hu H., Wei Y., Zhang Z., Lin S., Guo B., 2021. Swin transformer: Hierarchical vision transformer using shifted windows, in: Proceedings of the IEEE/CVF International Conference on Computer Vision. pp. 10012–10022.

[R84] Lu T., Zhang X., Huang Y., Guo D., Huang F., Xu Q., Hu Y., Ou-Yang L., Lin J., Yan Z., others, 2020. pFISTA-SENSE-ResNet for parallel MRI reconstruction. Journal of Magnetic Resonance 318, 106790. 32759045 10.1016/j.jmr.2020.106790

[R85] Lyu J., Tian Y., Cai Q., Wang C., Qin J., 2023. Adaptive channel-modulated personalized federated learning for magnetic resonance image reconstruction. Computers in Biology and Medicine 165, 107330. 37611426 10.1016/j.compbiomed.2023.107330

[R86] Lyu Q., Shan H., Steber C., Helis C., Whitlow C., Chan M., Wang G., 2020. Multi-contrast super-resolution MRI through a progressive network. IEEE transactions on medical imaging 39, 2738–2749. 32086201 10.1109/TMI.2020.2974858 PMC7673259

[R87] Maclaren J., Herbst M., Speck O., Zaitsev M., 2013. Prospective motion correction in brain imaging: a review. Magnetic resonance in medicine 69, 621–636. 22570274 10.1002/mrm.24314

[R88] Mardani M., Gong E., Cheng J.Y., Vasanawala S.S., Zaharchuk G., Xing L., Pauly J.M., 2018. Deep generative adversarial neural networks for compressive sensing MRI. IEEE transactions on medical imaging 38, 167–179. 30040634 10.1109/TMI.2018.2858752 PMC6542360

[R89] McMahan B., Moore E., Ramage D., Hampson S., y Arcas B.A., 2017. Communication-efficient learning of deep networks from decentralized data, in: Artificial Intelligence and Statistics. PMLR, pp. 1273–1282.

[R90] Mendrik A.M., Vincken K.L., Kuijf H.J., Breeuwer M., Bouvy W.H., De Bresser J., Alansary A., De Bruijne M., Carass A., El-Baz A., others, 2015. MRBrainS challenge: online evaluation framework for brain image segmentation in 3T MRI scans. Computational intelligence and neuroscience 2015, 1–1. 10.1155/2015/813696 PMC4680055 26759553

[R91] Meng Z., Guo R., Li Yudu, Guan Y., Wang T., Zhao Y., Sutton B., Li Yao, Liang Z.-P., 2021. Accelerating T2 mapping of the brain by integrating deep learning priors with lowrank and sparse modeling. Magnetic Resonance in Medicine 85, 1455–1467. 32989816 10.1002/mrm.28526

[R92] Menze B.H., Jakab A., Bauer S., Kalpathy-Cramer J., Farahani K., Kirby J., Burren Y., Porz N., Slotboom J., Wiest R., others, 2014. The multimodal brain tumor image segmentation benchmark (BRATS). IEEE transactions on medical imaging 34, 1993–2024. 25494501 10.1109/TMI.2014.2377694 PMC4833122

[R93] Mirza M., Osindero S., 2014. Conditional generative adversarial nets. arXiv preprint arXiv:1411.1784.

[R94] Murali S., Ding H., Adedeji F., Qin C., Obungoloch J., Asllani I., Anazodo U., Ntusi N.A., Mammen R., Niendorf T., others, 2023. Bringing MRI to low-and middle-income countries: directions, challenges and potential solutions. NMR in Biomedicine e4992. 37401341 10.1002/nbm.4992

[R95] Murugesan B., Ramanarayanan S., Vijayarangan S., Ram K., Jagannathan N.R., Sivaprakasam M., 2021. A deep cascade of ensemble of dual domain networks with gradient-based T1 assistance and perceptual refinement for fast MRI reconstruction. Computerized Medical Imaging and Graphics 91, 101942. 34087612 10.1016/j.compmedimag.2021.101942

[R96] Narnhofer D., Hammernik K., Knoll F., Pock T., 2019. Inverse GANs for accelerated MRI reconstruction, in: Wavelets and Sparsity XVIII. SPIE, pp. 381–392.

[R97] Pan S., Abouei E., Wynne J., Chang C.-W., Wang T., Qiu R.L., Li Y., Peng J., Roper J., Patel P., others, 2023a. Synthetic CT generation from MRI using 3D transformer-based denoising diffusion model. Medical Physics. 10.1002/mp.16847 PMC10994752 38011588

[R98] Pan S., Wang T., Qiu R.L., Axente M., Chang C.-W., Peng J., Patel A.B., Shelton J., Patel S.A., Roper J., others, 2023b. 2D medical image synthesis using transformer-based denoising diffusion probabilistic model. Physics in Medicine & Biology 68, 105004. 10.1088/1361-6560/acca5c PMC10160739 37015231

[R99] Pramanik A., Bhave S., Sajib S., Sharma S.D., Jacob M., 2023. Adapting model-based deep learning to multiple acquisition conditions: Ada-MoDL. Magnetic resonance in medicine 90, 2033–2051. 37332189 10.1002/mrm.29750 PMC10524947

[R100] Qiao X., Huang Y., Li W., 2023. MEDL-Net: A model-based neural network for MRI reconstruction with enhanced deep learned regularizers. Magnetic Resonance in Medicine 89, 2062–2075. 36656129 10.1002/mrm.29575

[R101] Qin C., Duan J., Hammernik K., Schlemper J., Küstner T., Botnar R., Prieto C., Price A.N., Hajnal J.V., Rueckert D., 2021. Complementary time-frequency domain networks for dynamic parallel MR image reconstruction. Magnetic Resonance in Medicine 86, 3274–3291. 34254355 10.1002/mrm.28917

[R102] Qin C., Schlemper J., Caballero J., Price A.N., Hajnal J.V., Rueckert D., 2018. Convolutional recurrent neural networks for dynamic MR image reconstruction. IEEE transactions on medical imaging 38, 280–290. 30080145 10.1109/TMI.2018.2863670

[R103] Qu B., Zhang J., Kang T., Lin J., Lin M., She H., Wu Q., Wang M., Zheng G., 2024. Radial magnetic resonance image reconstruction with a deep unrolled projected fast iterative soft-thresholding network. Computers in Biology and Medicine 168, 107707. 38000244 10.1016/j.compbiomed.2023.107707

[R104] Quan T.M., Nguyen-Duc T., Jeong W.-K., 2018. Compressed sensing MRI reconstruction using a generative adversarial network with a cyclic loss. IEEE transactions on medical imaging 37, 1488–1497. 29870376 10.1109/TMI.2018.2820120

[R105] Radmanesh A., Muckley M.J., Murrell T., Lindsey E., Sriram A., Knoll F., Sodickson D.K., Lui Y.W., 2022. Exploring the acceleration limits of deep learning variational network–based two-dimensional brain MRI. Radiology: Artificial Intelligence 4, e210313. 36523647 10.1148/ryai.210313 PMC9745443

[R106] Ravishankar S., Bresler Y., 2010. MR image reconstruction from highly undersampled kspace data by dictionary learning. IEEE transactions on medical imaging 30, 1028–1041. 21047708 10.1109/TMI.2010.2090538

[R107] Rohlfing T., Zahr N.M., Sullivan E.V., Pfefferbaum A., 2010. The SRI24 multichannel atlas of normal adult human brain structure. Human brain mapping 31, 798–819. 20017133 10.1002/hbm.20906 PMC2915788

[R108] Ronneberger O., Fischer P., Brox T., 2015. U-net: Convolutional networks for biomedical image segmentation, in: Medical Image Computing and Computer-Assisted Intervention–MICCAI 2015: 18th International Conference, Munich, Germany, October 5–9, 2015, Proceedings, Part III 18. Springer, pp. 234–241.

[R109] Ryu K., Lee J.-H., Nam Y., Gho S.-M., Kim H.-S., Kim D.-H., 2021. Accelerated multicontrast reconstruction for synthetic MRI using joint parallel imaging and variable splitting networks. Medical physics 48, 2939–2950. 33733464 10.1002/mp.14848

[R110] Safari M., Fatemi A., Archambault L., 2023a. MedFusionGAN: multimodal medical image fusion using an unsupervised deep generative adversarial network. BMC Medical Imaging 23, 203. 38062431 10.1186/s12880-023-01160-w PMC10704723

[R111] Safari M., Yang X., Fatemi A., 2024. MRI data consistency guided conditional diffusion probabilistic model for MR imaging acceleration, in: Medical Imaging 2024: Clinical and Biomedical Imaging. SPIE, pp. 202–205.

[R112] Safari M., Yang X., Fatemi A., Archambault L., 2023b. MRI motion artifact reduction using a conditional diffusion probabilistic model (MAR-CDPM). Medical Physics. 10.1002/mp.16844 38009583

[R113] Sandino C.M., Lai P., Vasanawala S.S., Cheng J.Y., 2021. Accelerating cardiac cine MRI using a deep learning-based ESPIRiT reconstruction. Magnetic Resonance in Medicine 85, 152–167. 32697891 10.1002/mrm.28420 PMC7722220

[R114] Schlemper J., Caballero J., Hajnal J.V., Price A., Rueckert D., 2017. A deep cascade of convolutional neural networks for MR image reconstruction, in: Information Processing in Medical Imaging: 25th International Conference, IPMI 2017, Boone, NC, USA, June 25–30, 2017, Proceedings 25. Springer, pp. 647–658.

[R115] Seo S., Luu H.M., Choi S.H., Park S.-H., 2022. Simultaneously optimizing sampling pattern for joint acceleration of multi-contrast MRI using model-based deep learning. Medical Physics 49, 5964–5980. 35678739 10.1002/mp.15790

[R116] Shaul R., David I., Shitrit O., Raviv T.R., 2020. Subsampled brain MRI reconstruction by generative adversarial neural networks. Medical Image Analysis 65, 101747. 32593933 10.1016/j.media.2020.101747

[R117] Shitrit O., Riklin Raviv T., 2017. Accelerated magnetic resonance imaging by adversarial neural network, in: Deep Learning in Medical Image Analysis and Multimodal Learning for Clinical Decision Support: Third International Workshop, DLMIA 2017, and 7th International Workshop, ML-CDS 2017, Held in Conjunction with MICCAI 2017, Québec City, QC, Canada, September 14, Proceedings 3. Springer, pp. 30–38.

[R118] Slavkova K.P., DiCarlo J.C., Wadhwa V., Kumar S., Wu C., Virostko J., Yankeelov T.E., Tamir J.I., 2023. An untrained deep learning method for reconstructing dynamic MR images from accelerated model-based data. Magnetic resonance in medicine 89, 1617–1633. 36468624 10.1002/mrm.29547 PMC9892348

[R119] Slipsager J.M., Glimberg S.L., Søgaard J., Paulsen R.R., Johannesen H.H., Martens P.C., Seth A., Marner L., Henriksen O.M., Olesen O.V., others, 2020. Quantifying the financial savings of motion correction in brain MRI: a model-based estimate of the costs arising from patient head motion and potential savings from implementation of motion correction. Journal of Magnetic Resonance Imaging 52, 731–738. 32144848 10.1002/jmri.27112

[R120] Sohl-Dickstein J., Weiss E., Maheswaranathan N., Ganguli S., 2015. Deep unsupervised learning using nonequilibrium thermodynamics, in: International Conference on Machine Learning. PMLR, pp. 2256–2265.

[R121] Sun K., Wang Q., Shen D., 2023. Joint Cross-Attention Network with Deep Modality Prior for Fast MRI Reconstruction. IEEE Transactions on Medical Imaging. 10.1109/TMI.2023.3314008 37695966

[R122] Sun L., Fan Z., Fu X., Huang Y., Ding X., Paisley J., 2019. A deep information sharing network for multi-contrast compressed sensing MRI reconstruction. IEEE Transactions on Image Processing 28, 6141–6153. 31295112 10.1109/TIP.2019.2925288

[R123] Sun L., Huang Y., Cai C., Ding X., 2017. Compressed sensing MRI using total variation regularization with K-space decomposition, in: 2017 IEEE International Conference on Image Processing (ICIP). IEEE, pp. 3061–3065.

[R124] Terpstra M.L., Maspero M., Verhoeff J.J., van den Berg C.A., 2023. Accelerated respiratory-resolved 4D-MRI with separable spatio-temporal neural networks. Medical physics 50, 5331–5342. 37527331 10.1002/mp.16643

[R125] Uecker M., Lai P., Murphy M.J., Virtue P., Elad M., Pauly J.M., Vasanawala S.S., Lustig M., 2014. ESPIRiT—an eigenvalue approach to autocalibrating parallel MRI: where SENSE meets GRAPPA. Magnetic resonance in medicine 71, 990–1001. 23649942 10.1002/mrm.24751 PMC4142121

[R126] Van Essen D.C., Smith S.M., Barch D.M., Behrens T.E., Yacoub E., Ugurbil K., Consortium W.-M.H., others, 2013. The WU-Minn human connectome project: an overview. Neuroimage 80, 62–79. 23684880 10.1016/j.neuroimage.2013.05.041 PMC3724347

[R127] Wang B., Lian Y., Xiong X., Zhou H., Liu Z., Zhou X., 2024. DCT-net: Dual-domain cross-fusion transformer network for MRI reconstruction. Magnetic Resonance Imaging 107, 69–79. 38237693 10.1016/j.mri.2024.01.007

[R128] Wang J., Awad M., Zhou R., Wang Z., Wang X., Feng X., Yang Y., Meyer C., Kramer C.M., Salerno M., 2024. High-resolution spiral real-time cardiac cine imaging with deep learning-based rapid image reconstruction and quantification. NMR in Biomedicine 37, e5051. 37926525 10.1002/nbm.5051 PMC12258077

[R129] Wang K., Tamir J.I., De Goyeneche A., Wollner U., Brada R., Yu S.X., Lustig M., 2022. High fidelity deep learning-based MRI reconstruction with instance-wise discriminative feature matching loss. Magnetic Resonance in Medicine 88, 476–491. 35373388 10.1002/mrm.29227

[R130] Wang S., Cheng H., Ying L., Xiao T., Ke Z., Zheng H., Liang D., 2020. DeepcomplexMRI: Exploiting deep residual network for fast parallel MR imaging with complex convolution. Magnetic resonance imaging 68, 136–147. 32045635 10.1016/j.mri.2020.02.002

[R131] Wang Z., Fang H., Qian C., Shi B., Bao L., Zhu L., Zhou J., Wei W., Lin J., Guo D., others, 2024. A faithful deep sensitivity estimation for accelerated magnetic resonance imaging. IEEE Journal of Biomedical and Health Informatics. 10.1109/JBHI.2024.3360128 38315596

[R132] Wang Z., Qian C., Guo D., Sun H., Li R., Zhao B., Qu X., 2022. One-dimensional deep low-rank and sparse network for accelerated MRI. IEEE Transactions on Medical Imaging 42, 79–90. 36044484 10.1109/TMI.2022.3203312

[R133] Wang Z., She H., Zhang Y., Du Y.P., 2023. Parallel non-Cartesian spatial-temporal dictionary learning neural networks (stDLNN) for accelerating 4D-MRI. Medical image analysis 84, 102701. 36470148 10.1016/j.media.2022.102701

[R134] Wen J., Ahmad R., Schniter P., 2023. A conditional normalizing flow for accelerated multi-coil MR imaging. Proceedings of machine learning research 202, 36926. 38084206 PMC10712023

[R135] Wu Y., Ma Y., Liu J., Du J., Xing L., 2019. Self-attention convolutional neural network for improved MR image reconstruction. Information sciences 490, 317–328. 32817993 10.1016/j.ins.2019.03.080 PMC7430761

[R136] Wu Z., Liao W., Yan C., Zhao M., Liu G., Ma N., Li X., 2023. Deep learning based MRI reconstruction with transformer. Computer Methods and Programs in Biomedicine 233, 107452. 36924533 10.1016/j.cmpb.2023.107452

[R137] Xiang L., Chen Y., Chang W., Zhan Y., Lin W., Wang Q., Shen D., 2018. Ultra-fast t2-weighted mr reconstruction using complementary t1-weighted information, in: Medical Image Computing and Computer Assisted Intervention–MICCAI 2018: 21st International Conference, Granada, Spain, September 16–20, 2018, Proceedings, Part I. Springer, pp. 215–223. 10.1007/978-3-030-00928-1_25 PMC6430217 30906934

[R138] Xie H., Lei Y., Wang T., Roper J., Dhabaan A.H., Bradley J.D., Liu T., Mao H., Yang X., 2022. Synthesizing high-resolution magnetic resonance imaging using parallel cycleconsistent generative adversarial networks for fast magnetic resonance imaging. Medical Physics 49, 357–369. 34821395 10.1002/mp.15380 PMC11699524

[R139] Xie Y., Li Q., 2022. A Review of Deep Learning Methods for Compressed Sensing Image Reconstruction and Its Medical Applications. Electronics 11, 586. 10.3390/electronics11040586

[R140] Xu J., Zu T., Hsu Y.-C., Wang X., Chan K.W., Zhang Y., 2024. Accelerating CEST imaging using a model-based deep neural network with synthetic training data. Magnetic Resonance in Medicine 91, 583–599. 37867413 10.1002/mrm.29889

[R141] Xue Y., Yu J., Kang H.S., Englander S., Rosen M.A., Song H.K., 2012. Automatic coil selection for streak artifact reduction in radial MRI. Magnetic Resonance in Med 67, 470–476. 10.1002/mrm.23023 21656562

[R142] Yaman B., Gu H., Hosseini S.A.H., Demirel O.B., Moeller S., Ellermann J., Uğurbil K., Akçakaya M., 2022. Multi-mask self-supervised learning for physics-guided neural networks in highly accelerated magnetic resonance imaging. NMR in Biomedicine 35, e4798. 35789133 10.1002/nbm.4798 PMC9669191

[R143] Yaman B., Hosseini S.A.H., Moeller S., Ellermann J., Uğurbil K., Akçakaya M., 2020. Selfsupervised learning of physics-guided reconstruction neural networks without fully sampled reference data. Magnetic resonance in medicine 84, 3172–3191. 32614100 10.1002/mrm.28378 PMC7811359

[R144] Yan Y., Yang T., Zhao X., Jiao C., Yang A., Miao J., 2023. DC-SiamNet: Deep contrastive Siamese network for self-supervised MRI reconstruction. Computers in Biology and Medicine 167, 107619. 37925909 10.1016/j.compbiomed.2023.107619

[R145] Yang G., Yu S., Dong H., Slabaugh G., Dragotti P.L., Ye X., Liu F., Arridge S., Keegan J., Guo Y., others, 2017. DAGAN: deep de-aliasing generative adversarial networks for fast compressed sensing MRI reconstruction. IEEE transactions on medical imaging 37, 1310–1321. 10.1109/TMI.2017.2785879 29870361

[R146] Yang Q., Liu Y., Chen T., Tong Y., 2019. Federated machine learning: Concept and applications. ACM Transactions on Intelligent Systems and Technology (TIST) 10, 1–19.

[R147] Yang Y., Sun J., Li H., Xu Z., 2016. Deep ADMM-Net for compressive sensing MRI, in: Proceedings of the 30th International Conference on Neural Information Processing Systems. pp. 10–18.

[R148] Yiasemis G., Sánchez C.I., Sonke J.-J., Teuwen J., 2024. On retrospective k-space subsampling schemes for deep MRI reconstruction. Magnetic Resonance Imaging 107, 33–46. 38184093 10.1016/j.mri.2023.12.012

[R149] Yoon M.A., Gold G.E., Chaudhari A.S., 2023. Accelerated Musculoskeletal Magnetic Resonance Imaging. Magnetic Resonance Imaging jmri.29205. 10.1002/jmri.29205 38156716

[R150] Zbontar J., Knoll F., Sriram A., Murrell T., Huang Z., Muckley M.J., Defazio A., Stern R., Johnson P., Bruno M., others, 2018. fastMRI: An open dataset and benchmarks for accelerated MRI. arXiv preprint arXiv:1811.08839.

[R151] Zhao X., Yang T., Li B., Zhang X., 2023. SwinGAN: A dual-domain Swin Transformer-based generative adversarial network for MRI reconstruction. Computers in Biology and Medicine 153, 106513. 36603439 10.1016/j.compbiomed.2022.106513

[R152] Zhou B., Schlemper J., Dey N., Salehi S.S.M., Sheth K., Liu C., Duncan J.S., Sofka M., 2022. Dual-domain self-supervised learning for accelerated non-Cartesian MRI reconstruction. Medical Image Analysis 81, 102538. 35926336 10.1016/j.media.2022.102538

[R153] Zhou Z., Han F., Ghodrati V., Gao Y., Yin W., Yang Y., Hu P., 2019. Parallel imaging and convolutional neural network combined fast MR image reconstruction: Applications in low-latency accelerated real-time imaging. Medical physics 46, 3399–3413. 31135966 10.1002/mp.13628 PMC9555742

[R154] Zhu J.-Y., Park T., Isola P., Efros A.A., 2017. Unpaired image-to-image translation using cycle-consistent adversarial networks, in: Proceedings of the IEEE International Conference on Computer Vision. pp. 2223–2232.

[R155] Zufiria B., Qiu S., Yan K., Zhao R., Wang R., She H., Zhang C., Sun B., Herman P., Du Y., others, 2022. A feature-based convolutional neural network for reconstruction of interventional MRI. NMR in Biomedicine 35, e4231. 31856431 10.1002/nbm.4231

